# Phylogenetic relationships and codon usage bias amongst cluster K mycobacteriophages

**DOI:** 10.1093/g3journal/jkab291

**Published:** 2021-08-17

**Authors:** Adele Crane, Cyril J Versoza, Tiana Hua, Rohan Kapoor, Lillian Lloyd, Rithik Mehta, Jueliet Menolascino, Abraham Morais, Saige Munig, Zeel Patel, Daniel Sackett, Brandon Schmit, Makena Sy, Susanne P Pfeifer

**Affiliations:** 1 School of Life Sciences, Arizona State University, Tempe, AZ 85281, USA; 2 Center for Evolution and Medicine, Arizona State University, Tempe, AZ 85281, USA; 3 Center for Mechanisms of Evolution, Arizona State University, Tempe, AZ 85281, USA

**Keywords:** mycobacteriophages, cluster K, *de novo* assembly, genome annotation, phylogeny, codon usage bias

## Abstract

Bacteriophages infecting pathogenic hosts play an important role in medical research, not only as potential treatments for antibiotic-resistant infections but also offering novel insights into pathogen genetics and evolution. A prominent example is cluster K mycobacteriophages infecting *Mycobacterium tuberculosis*, a causative agent of tuberculosis in humans. However, as handling *M. tuberculosis* as well as other pathogens in a laboratory remains challenging, alternative nonpathogenic relatives, such as *Mycobacterium smegmatis*, are frequently used as surrogates to discover therapeutically relevant bacteriophages in a safer environment. Consequently, the individual host ranges of the majority of cluster K mycobacteriophages identified to date remain poorly understood. Here, we characterized the complete genome of Stinson, a temperate subcluster K1 mycobacteriophage with a siphoviral morphology. A series of comparative genomic analyses revealed strong similarities with other cluster K mycobacteriophages, including the conservation of an immunity repressor gene and a toxin/antitoxin gene pair. Patterns of codon usage bias across the cluster offered important insights into putative host ranges in nature, highlighting that although all cluster K mycobacteriophages are able to infect *M. tuberculosis*, they are less likely to have shared an evolutionary infection history with *Mycobacterium leprae* (underlying leprosy) compared to the rest of the genus’ host species. Moreover, subcluster K1 mycobacteriophages are able to integrate into the genomes of *Mycobacterium abscessus* and *Mycobacterium marinum—*two bacteria causing pulmonary and cutaneous infections which are often difficult to treat due to their drug resistance.

## Introduction

First discovered by Frederick Twort and Félix d'Herelle in the early 20th century, bacteriophages (*i.e.*, viruses that infect bacteria) are one of the most abundant biological entities on our planet (Rohwer 2003). Their study can provide important insights, not only into their own genetics and evolution (Hatfull 2010) but also into that of their (often) pathogenic hosts, many of which are intimately linked to human health and disease (Hatfull 2012). Due to the challenges involved in the handling of pathogenic strains in laboratories, fast-growing nonpathogenic strains (*e.g.*, *Mycobacterium smegmatis* mc^2^155) are frequently used as surrogates (Mizuguchi 1984). These strains are also natural choices for course-based undergraduate research experiences, such as Howard Hughes Medical Institute’s Science Education Alliance—Phage Hunters Advancing Genomics and Evolutionary Science (HHMI SEA-PHAGES; Hatfull 2006). Undergraduate researchers involved in the program—now running in its 13th year—have isolated ∼18,000 bacteriophages, including 2,015 mycobacteriophages (https://phagesdb.org; last accessed August 3, 2021; Russell and Hatfull 2017). Based on the similarity in the nucleotide sequence, these can be grouped into 29 clusters (A-Z, AA, AB, and AC) which are further divided into several subclusters with common genomic architectures, as well as a few individual outliers without any close relatives yet defined (Hatfull 2010; Pope *et al.* 2011b). Among these, cluster K mycobacteriophages are of particular interest to the scientific community due to their ability to infect *Mycobacterium* *tuberculosis* (Pope *et al.* 2011a)—the primary bacterium underlying tuberculosis which causes more than 1 million deaths per year (World Health Organization 2020). Cluster K mycobacteriophages have successfully been used to diagnose tuberculosis infections (*e.g.*, Jain *et al.* 2011), genetically manipulate *M. tuberculosis* (*e.g.*, by transferring foreign DNA via shuttle plasmids; Jacobs *et al.* 1987), and assess drug susceptibility (*e.g.*, Jacobs *et al.* 1993; Piuri *et al.* 2009). Moreover, they hold great promise for the treatment of drug-resistant tuberculosis strains via bacteriophage therapy, as well as prophylaxis aiding in the prevention of *M. tuberculosis* infections (see review by Allué-Guardia *et al.* 2021 and references therein). Although studies remain limited to date (Sula *et al.* 1981; Peng *et al.* 2009; and see review by Azimi *et al.* 2019), research into these medical and therapeutic applications will likely accelerate in the near future, spearheaded by the recently FDA-approved Center for Innovative Phage Applications and Therapeutics.

Many cluster K mycobacteriophages have the ability to infect a diverse range of hosts—from slow growing (*e.g.*, *M. tuberculosis*) to fast growing (*e.g.*, *M. smegmatis*) mycobacteria (Pope *et al.* 2011a), yet detailed insights into individual host ranges remain largely lacking due to the fact that the majority of known mycobacteriophages were isolated using *M. smegmatis* mc^2^155. As most bacteriophages utilize the translational machinery of their host, their protein synthesis is more efficient if their codon usage patterns (*i.e.*, preferences in the usage of synonymous codons) are in agreement with those of their hosts (Carbone 2008; Lucks *et al.* 2008; Esposito *et al.* 2016). Therefore, a better understanding of bacteriophage codon usage biases can provide important clues toward both their mycobacterial host range in nature (Hassan *et al.* 2009) as well as candidates for future bacteriophage therapies. Here, we characterize the complete genome sequence of Stinson, a temperate subcluster K1 mycobacteriophage, and, through computational analysis of codon usage bias patterns, infer putative host ranges of 129 genomically characterized cluster K mycobacteriophages to aid future medical and therapeutic investigations.

## Materials and methods

Sample collection, isolation, purification, and amplification of Stinson followed the HHMI SEA-PHAGES Phage Discovery Guide (https://seaphagesphagediscoveryguide.helpdocsonline.com/home; last accessed August 3, 2021); sequencing, *de novo* assembly, and genome annotation followed the HHMI SEA-PHAGES Bioinformatics Guide (https://seaphagesbioinformatics.helpdocsonline.com/home; last accessed August 3, 2021).

### Sample collection and isolation

Stinson was obtained from a soil sample collected at the Hope College Pine Grove (42.787471 N, 86.102762 W) on a warm, humid morning. The sample was enriched by adding a bacterial host culture of *M. smegmatis* mc^2^155, submerging in 7H9 liquid medium [Middlebrook 7H9 broth base, 0.5% glycerol 10% AD supplement (145 mM NaCl, 5% Albumin Fraction V, 2% dextrose), 50 μg/mL carbenicillin stock, 10 μg/mL cycloheximide stock, 1 mM CaCl_2_], and incubating at 32°C for 2–5 days. After incubation, the enriched culture was centrifuged and filtered through a 0.22-μm filter. A spot test was performed by adding 250 μL of *M. smegmatis* mc^2^155 culture to 3 mL of molten top agar (1 mM CaCl_2_, 7H9 liquid media, agar), then immediately plating on an L-Agar plate [Luria broth base (10 g peptone, 5 g yeast extract, 10 g NaCl), agar, 50 μg/mL CB, 10 μg/mL CHX]. Ten microliters of bacteriophage filtrate was transferred to the plate, which was incubated at 32°C for 24–48 h before checking for plaques.

### Purification and amplification

Bacteriophages were purified by selecting a single, well-isolated (1.5 cm apart) plaque to resuspend in phage buffer (10 mM pH 7.5 Tris, 10 mM MgSO_4_, 68 mM NaCl, 1 mM CaCl_2_). A series of 10-fold dilutions were performed and each dilution was dispensed into a tube containing 250 μL of host bacteria. The sample was incubated at room temperature for 10 min, then mixed with 3 mL of molten top agar, and immediately plated on an agar plate. The plates were incubated at 32°C for 24–48 h. This step was repeated until countable plates showed plaques of similar morphology that suggested that each grew from a single bacteriophage.

Bacteriophage lysate was harvested by flooding a plate containing a large number of purified bacteriophage plaques with 8 mL of phage buffer. The plate was set at room temperature for an hour, and then lysate was collected and filtered through a 0.22-μm filter. Tenfold serial dilutions were made with the bacteriophage lysate and then plated for a spot titer assay. Based on the spot titer results, a full titer assay was completed and the bacteriophage lysate titer was calculated to be 4.2 × 10^10^ PFU/mL.

### Sequencing, *de novo* assembly, and genome annotation

Genomic DNA, extracted using a Promega Wizard^®^ DNA Clean-Up kit, was used to prepare a single-indexed (TruSeq) sequencing library, which was subsequently sequenced on an Illumina MiSeq instrument, resulting in 2,197,235 high-quality single-end 150-bp reads (>5500X coverage; Supplementary Figure S1). Following Russell (2018), reads were *de novo* assembled using Newbler v.2.9, resulting in a fully assembled genome (length: 59,918 base pairs). The resulting assembly was checked for completeness, accuracy, and genomic termini using Consed v.29.0 (Gordon *et al.* 1998).

The genome of bacteriophage Stinson was annotated using DNA Master v.5.23.6 following Pope and Jacobs-Sera (2018), along with GLIMMER v.3.02 (Delcher *et al.* 1999) and GeneMark v.2.5 (Lukashin and Borodovsky 1998) embedded within. Start codons of putative genes were chosen to maximize coding potential (as reported by GeneMark) and confirmed using Starterator v.1.2 reports from closely related bacteriophages (https://seaphages.org/software/#Starterator; last accessed August 3, 2021). Predicted genes as well as any gaps larger than 10 bp were blasted both within the DNA Master environment using BLASTp v.2.9 (Altschul *et al.* 1990) as well as on NCBI (https://blast.ncbi.nlm.nih.gov/Blast.cgi; last accessed August 3, 2021). Aragorn v.1.1 (embedded in DNA Master) was utilized to locate tRNAs in the genome and Aragorn v1.2.38 (Laslett and Canback 2004) was applied for improved recognition of the ends of the identified tRNA. In addition, the software tRNAscan-SE v.2.0 (Lowe and Eddy 1997) was employed to search for noncanonical tRNAs. The protein product sequence from DNA Master was processed on HHPred (Söding *et al.* 2005), as well as on NCBI BLAST, utilizing the nonredundant protein sequences database (Marchler-Bauer *et al.* 2015), which assisted in the determination of putative gene functions. Genes with no known function were refined through TMHMM v.2.0 (Krogh *et al.* 2001) and SOSUI v.1.11 (Hirokawa *et al.* 1998) to predict membrane proteins. Lastly, Phamerator (Cresawn *et al.* 2011) was used to assess synteny among closely related bacteriophages. All software was executed using default settings.

### Comparative genomic analyses

To characterize phylogenetic relationships and patterns of codon usage bias, genomic data for 128 *Mycobacterium* cluster K bacteriophages was downloaded from the NCBI Sequence Read Archive (Supplementary Table S1). A multiple-sequence alignment was generated with MAFFT v.7 (Katoh and Standley 2013) through the online EMBL-EBI bioinformatics toolkit (Madeira *et al.* 2019) and used to construct a neighbor-joining tree in MEGA X (Kumar *et al.* 2018) using a bootstrap test of phylogeny with 10,000 replicates. Closely related bacteriophages were then visually compared using Phamerator (Cresawn *et al.* 2011). In addition, dot plots, generated using Gepard v.1.40 (Krumsiek *et al.* 2007), were used to compare nucleotide sequence relatedness among the bacteriophages. Pairwise Average Nucleotide Identities (ANIs) were calculated using the DNA Master v.5.23.6 Genome Comparison Tool and plotted using the heatmap.2 function in R v.4.0.2. To determine codon usage bias within and across genomes, the COdon Usage Similarity INdex (COUSIN_59_) was calculated for each of the 129 mycobacteriophages (Supplementary Table S1) across 14 putative mycobacterial host species (Supplementary Table S2) using the software COUSIN (Bourret *et al.* 2019). All software was executed using default settings.

### Identification of prophages within putative bacterial host genomes

PHASTER (https://phaster.ca; last accessed August 3, 2021; Arndt *et al.* 2016) was utilized to identify prophage sequences within the 14 putative mycobacterial host species (Supplementary Table S2). Next, MUSCLE v.3.8 (https://www.ebi.ac.uk/Tools/msa/muscle/; last accessed August 3, 2021; Edgar 2004) was used to study the evolutionary relationship between the immunity repressor gene of Stinson and the subcluster K1 mycobacteriophages able to integrate in these bacterial hosts. All software was executed using default settings.

## Results and discussion

**Figure 1:**
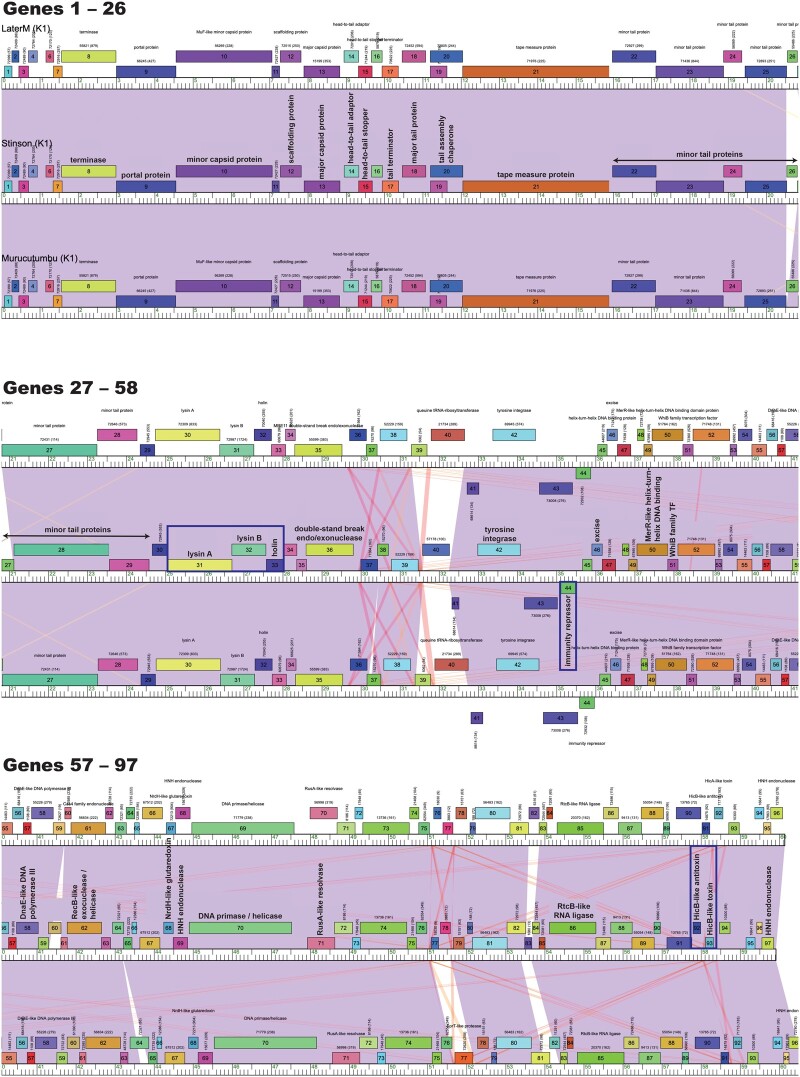
Phamerator map of the whole genomes of Stinson and two closely related *Mycobacterium* bacteriophages, LaterM (Gaballa *et al.* 2019) and Murucutumbu (Pope *et al.* 2015). In this Phamerator map, protein-coding genes with their putative functional assignments (if available) are displayed above or below a ruler, signifying genes on forward or reverse strands, respectively. The numbers shown above each gene indicate the protein family (pham) and, in parenthesis, the number of members in the pham family. Coloring between genomes represents nucleotide similarity with areas of highest similarity shown in purple (BLAST *e*-value = 0), followed by red (BLAST *e*-value of ∼10^−4^) and white (no significant similarity). Stinson’s lysis cassette (lysin A and lysin B), holin, the immunity repressor gene, and the TA system (highlighted by blue boxes) show strong similarities between the three mycobacteriophages.

Stinson is a cluster K mycobacteriophage with a *Siphoviridae* morphology (*i.e.*, nonenveloped with an icosahedral head and a noncontractile tail) and a temperate lifestyle. The fully sequenced and annotated genome is 59,918 base pairs long, with a GC-content of 66.6%—similar to that of its host, *M. smegmatis* mc^2^ 155 (67.4%). Stinson’s genome contains 96 tightly packed protein-coding genes as well as a single tRNA, corresponding to a gene density of 1.60 genes/kb (Figure 1)—within the previously reported range for cluster K mycobacteriophages (1.34–1.74 genes/kb; Supplementary Table S1). Synteny is preserved with other cluster K mycobacteriophages, with the left arm of the genome encoding for structural and assembly proteins (*i.e.*, terminase, portal protein, MuF-like minor capsid protein, scaffolding protein, major capsid protein, head-to-tail adaptor, head-to-tail stopper, tail terminator, major tail protein, tail assembly chaperones expressed via a translational frameshift (Xu et al. 2004), tape measure protein, and eight minor tail protein subunits), followed by the lysis cassette (lysin A and lysin B), holin, and genes responsible for integration into the host (Figure 1). The right arm of the genome contains nonstructural genes (Hatfull 2014), several of which are of unknown function. An important exception is the immunity repressor (gene 44), a downregulator of lytic gene expression, suggesting that Stinson might exhibit lysogenic maintenance and superinfection immunity (Petrova *et al.* 2015). Also of note is the toxin/antitoxin (TA) system (genes 92 and 93) which shows similarity to *hicAB*, a TA system found in *Pseudomonas aeruginosa* and *Escherichia coli* (Yamaguchi and Inouye 2011; Li *et al.* 2016). Overall, 41 out of the 96 genes could be assigned a putative function and an additional three genes were identified as membrane proteins. Similar to other cluster K mycobacteriophages, Stinson encodes a tRNA^trp^ (gene 5) as well as an RtcB-like RNA ligase (gene 86), likely serving to protect bacteriophages against tRNA cleavage by the host (Labrie *et al.* 2010; Pope *et al.* 2011a).

**Figure 2:**
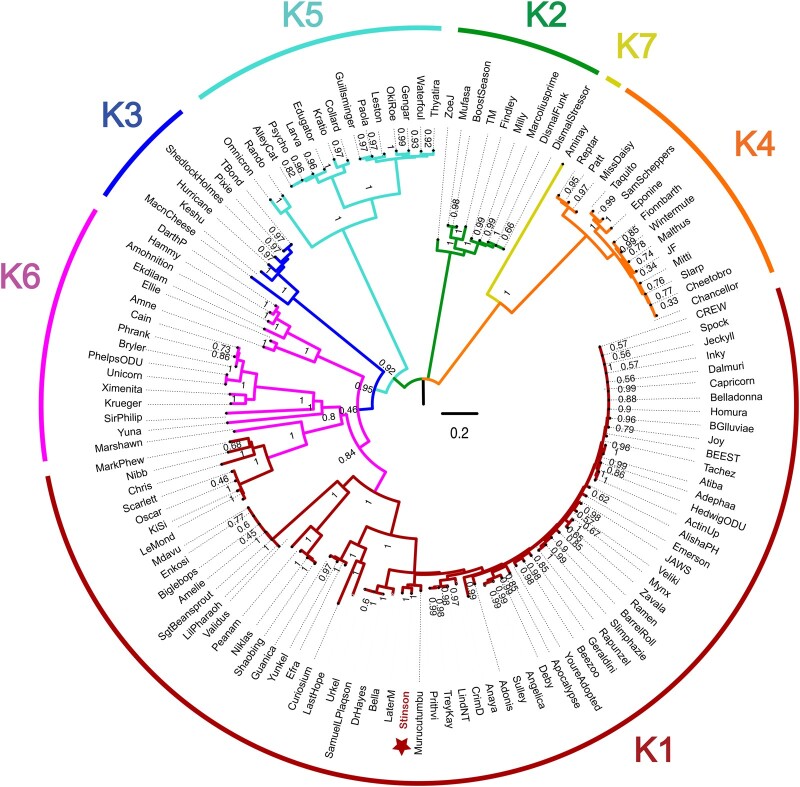
Neighbor-joining tree generated using a multiple-sequence alignment of 129 *Mycobacterium* bacteriophage cluster K genomes (Supplementary Table S1) with 10,000 bootstrap replicates. Colors highlight membership in subclusters K1–K7.

**Figure 3:**
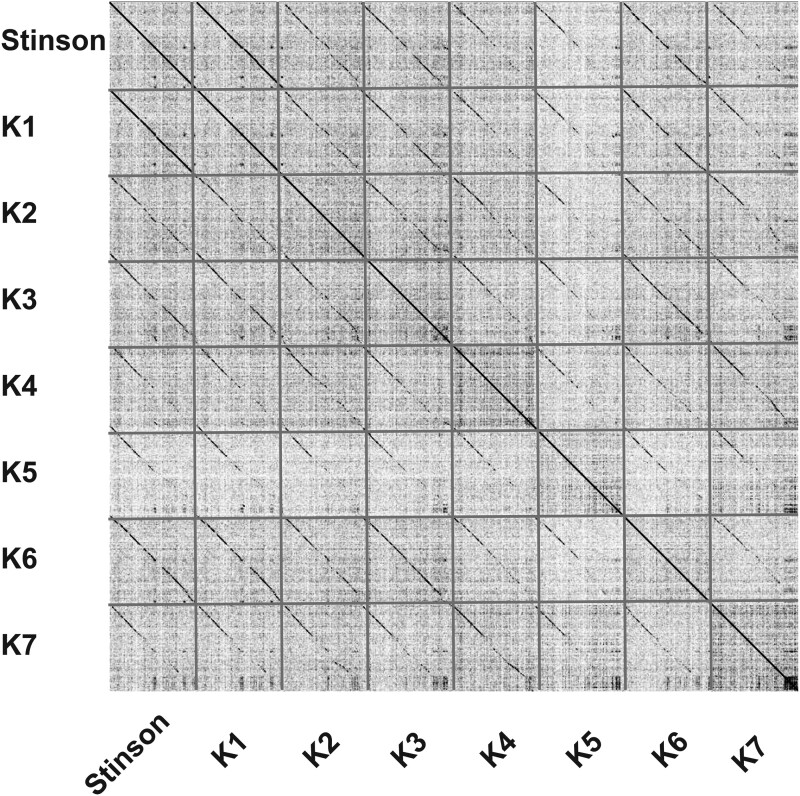
Dot plot of Stinson and one representative from each of the *Mycobacterium* bacteriophage cluster K subclusters: LaterM (K1), TM4 (K2), Pixie (K3), Cheetobro (K4), Collard (K5), Unicorn (K6), and Aminay (K7). Detailed information regarding each mycobacteriophage’s genome is provided in Supplementary Table S1.

**Figure 4:**
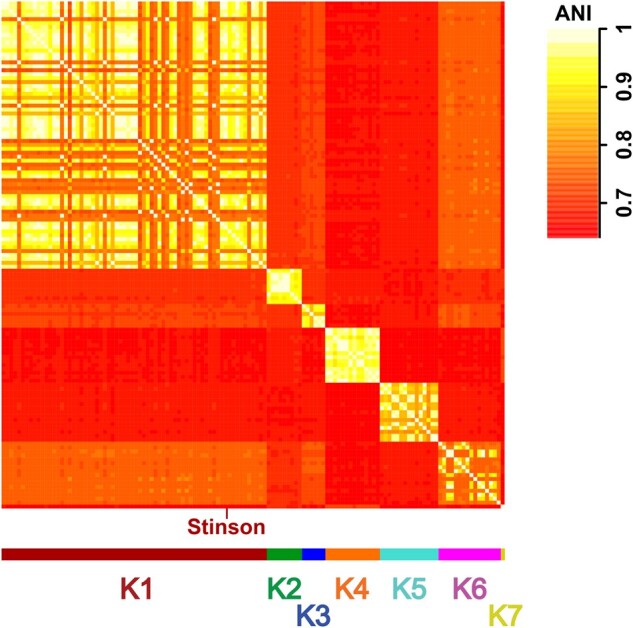
Heatmap of ANI values of the 129 *Mycobacterium* bacteriophage genomes (Supplementary Table S1). Colors highlight membership in subclusters K1–K7.

To characterize phylogenetic relationships as well as patterns of codon usage bias amongst cluster K mycobacteriophages, comparative analyses focused on Stinson and 128 previously characterized mycobacteriophages, including 67 members of subcluster K1, 9 of subcluster K2, 6 of subcluster K3, 14 of subcluster K4, 15 of subcluster K5, 16 of subcluster K6, and 1 of subcluster K7. The constructed neighbor-joining tree suggests that Stinson is a member of the K1 subcluster—a subcluster that shares a common ancestor with Marshawn (GenBank accession number: MN284895), a member of the paraphyletic subcluster K6 (Figure 2). This classification is supported by both the dot plot analysis (Figure 3 and Supplementary Figures S2–S8) as well as the pairwise ANI (Figure 4), which also highlights the strong relatedness between Stinson and other cluster K1 mycobacteriophages on the sequence level. Among the 67 subcluster K1 mycobacteriophages, Stinson is most closely related to LaterM (Gaballa *et al.* 2019) and Murucutumbu (Pope *et al.* 2015), with a nucleotide percent identity of 98.34% and 97.73%, respectively.

**Figure 5:**
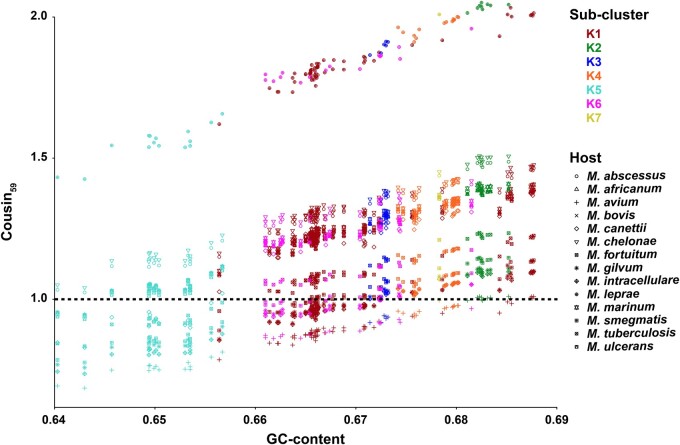
COdon Usage Similarity INdex (COUSIN_59_) of 129 *Mycobacterium* bacteriophage genomes (Supplementary Table S1) across 14 *Mycobacterium* host species (Supplementary Table S2), ordered by GC-content of the mycobacteriophage genomes. Colors highlight membership in subclusters K1–K7; shapes refer to the *Mycobacterium* host species.

Patterns of codon usage bias amongst the 129 mycobacteriophages highlight strong similarities to the preferred usage of synonymous codons of 13 out of the 14 putative *Mycobacterium* host species (Figure 5), as may be expected from similarities in genomic GC-content (64–69%; Supplementary Tables S1 and S2). An important exception is the bacterium *Mycobacterium leprae* (GC-content: 57.8%; Supplementary Table S2), the primary causative agent of leprosy in humans. These results are in agreement with more limited experimental evidence highlighting that all cluster K mycobacteriophages are able to infect *M. tuberculosis* (Pope *et al.* 2011a) but suggest that they are less likely to have shared an evolutionary infection history with *M. leprae* compared to the rest of the genus’ host species.

**Figure 6:**
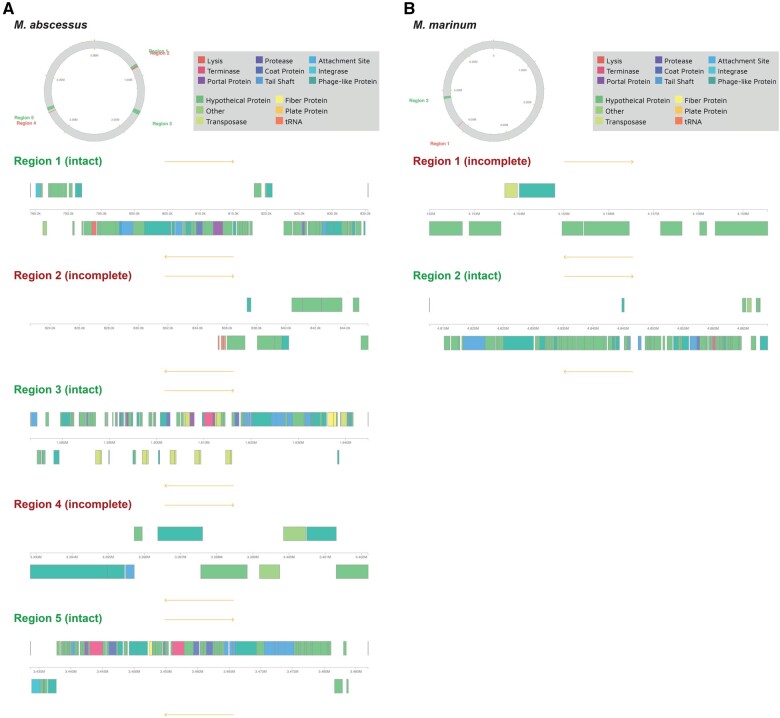
Identification of prophages within (A) *M. abscessus* and (B) *M. marinum*. Regions displayed in green contain an intact and regions in red an incomplete prophage. Within a region, phage-like proteins are shown in dark green above or below a ruler, signifying proteins on forward or reverse strands, respectively.

To investigate whether Stinson or any closely related subcluster K1 mycobacteriophages might exhibit lysogenic properties, prophage sequences within the genomes of the putative hosts (Supplementary Table S2) were computationally predicted. The genome of 3 out of the 14 putative host species include intact prophages (Supplementary Table S3): *Mycobacterium abscessus* (GC-content: 64.2%) contains three intact and two incomplete prophages, *Mycobacterium* *marinum* (GC-content: 65.2%) contains one intact and one incomplete prophage, and *M. smegmatis* (GC-content: 67.4%) contains one intact and nine incomplete prophages. Although none of the prophages contained in the genome of *M. smegmatis* were predicted to have arisen from the integration of mycobacteriophages, the genomes of *M. abscessus* (Figure 6A) and *M. marinum* (Figure 6B) harbor prophages from several subcluster K1 (Adephagia, Amelie, Beezoo, and LastHope) as well as other cluster K mycobacteriophages (Supplementary Table S3). Given the similarity between Stinson and the subcluster K1 mycobacteriophages (see Figure 2 for a genome-wide representation and Supplementary Figure S9 for the immunity repressor gene specifically), it is expected that Stinson will also be able to integrate in these bacterial hosts. As *M. abscessus* and *M. marinum* play important roles in human health and disease, causing pulmonary (Winthrop and Roy 2020) and cutaneous (Aubry *et al.* 2000) infections which are often difficult to treat due to drug resistance, cluster K mycobacteriophages might provide important future avenues toward improved medical and therapeutic management strategies.

## Data availability

Supplementary Figure S1 displays the quality control of Stinson’s raw reads. Supplementary Figures S2–S8 show the dot plots of Stinson and mycobacteriophages from subclusters K1–K7, respectively. Supplementary Figure S9 displays the multiple-sequence alignment of the immunity repressor gene of Stinson and the subcluster K1 mycobacteriophages able to integrate in the putative bacterial hosts. Mycobacteriophages and putative mycobacterial hosts included in the comparative analyses are listed in Supplementary Tables S1 and S2, respectively. Prophages contained within the mycobacterial genomes are listed in Supplementary Table S3. Whole-genome sequence data are available through the NCBI Sequence Read Archive (BioProject accession number PRJNA488469). The annotated genome assembly is available through NCBI GenBank (accession number MZ355721).

Supplementary material is available at *G3* online.

## Supplementary Material

jkab291_Supplementary_Data
