# Supplementary material for: Phylogenetic relationships and codon usage bias amongst cluster K mycobacteriophages
Source: G3 (Bethesda). 2021 Aug 17;11(11):jkab291. doi: 10.1093/g3journal/jkab291 (PMC8527509; doi:10.1093/g3journal/jkab291)
Supplement: jkab291_Supplementary_Data [file jkab291_supplementary_data.zip › GENETICS-G3-2021-402777-s01.docx]

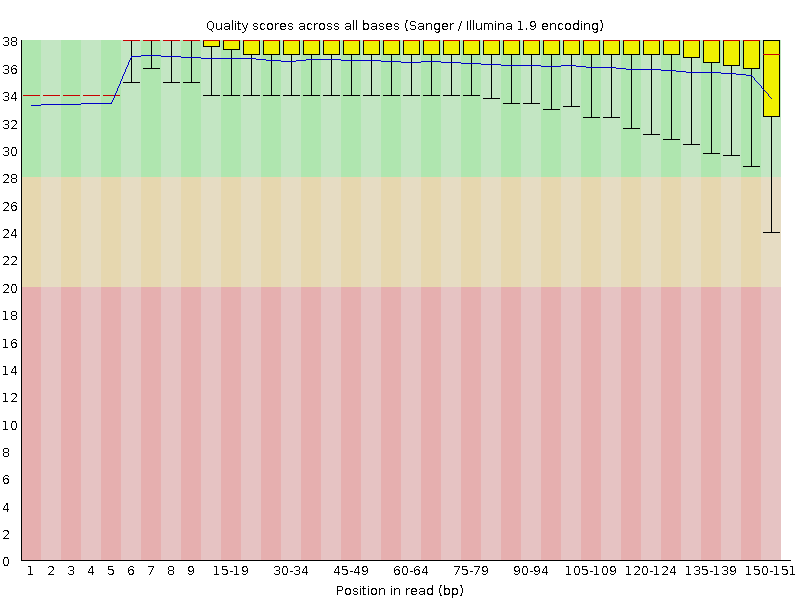


**Figure S1**. Quality control of Stinson's raw reads. The box-and-whiskers plot is showing Phred-scaled base quality scores (y-axis) for the 150 sequenced bases (x-axis). Red lines display median values; blue lines mean values.

**Figure S2**. Dot plot of Stinson (indicated by a red star) and mycobacteriophages from sub-cluster K1. Detailed information regarding each bacteriophage’s genome is provided in Table S1.

**Figure S3**. Dot plot of Stinson (indicated by a red star) and mycobacteriophages from sub-cluster K2. Detailed information regarding each bacteriophage’s genome is provided in Table S1.

**Figure S4**. Dot plot of Stinson (indicated by a red star) and mycobacteriophages from sub-cluster K3. Detailed information regarding each bacteriophage’s genome is provided in Table S1.

**Figure S5**. Dot plot of Stinson (indicated by a red star) and mycobacteriophages from sub-cluster K4. Detailed information regarding each bacteriophage’s genome is provided in Table S1.

**Figure S6**. Dot plot of Stinson (indicated by a red star) and mycobacteriophages from sub-cluster K5. Detailed information regarding each bacteriophage’s genome is provided in Table S1.

**Figure S7**. Dot plot of Stinson (indicated by a red star) and mycobacteriophages from sub-cluster K6. Detailed information regarding each bacteriophage’s genome is provided in Table S1.

**Figure S8**. Dot plot of Stinson (indicated by a red star) and Aminay, a mycobacteriophage from sub-cluster K7. Detailed information regarding each bacteriophage’s genome is provided in Table S1.


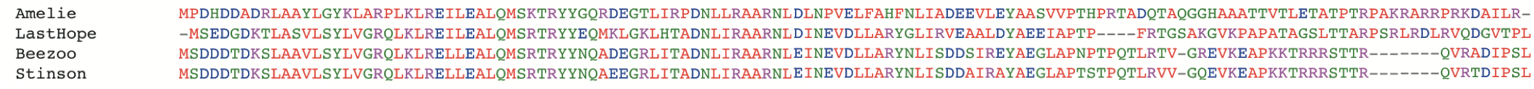


**Figure S9**. Multiple sequence alignment of the immunity repressor gene of Stinson and the sub-cluster K1 bacteriophages able to integrate in the putative bacterial hosts (Table S3).
