# Supplementary material for: Phylogenetic relationships and codon usage bias amongst cluster K mycobacteriophages
Source: G3 (Bethesda). 2021 Aug 17;11(11):jkab291. doi: 10.1093/g3journal/jkab291 (PMC8527509; doi:10.1093/g3journal/jkab291)
Supplement: jkab291_Supplementary_Data [file jkab291_supplementary_data.zip › GENETICS-G3-2021-402777-s02.docx]

**Table S1. Mycobacteriophages included in the comparative analyses.**

| **Phage Name** | **Sub-cluster** | **Length (bp)** | **GC-content** | **# ORFs** | **# tRNAs** | **Accession #** | **Reference** |
| --- | --- | --- | --- | --- | --- | --- | --- |
| ActinUp | K1 | 59,812 | 66.6 | 96 | 1 | MH051246 | unpublished |
| Adephagia | K1 | 59,646 | 66.6 | 94 | 1 | JF704105 | Hatfull 2012a |
| Adonis | K1 | 60,031 | 66.7 | 95 | 1 | MH001453 | unpublished |
| AlishaPH | K1 | 57,034 | 66.6 | 88 | 1 | MH077577 | unpublished |
| Amelie | K1 | 56,439 | 67.1 | 77 | 0 | KX808132 | Pope *et al.* 2015 |
| Anaya | K1 | 60,835 | 66.4 | 98 | 1 | JF704106 | Pope *et al.* 2015 |
| Angelica | K1 | 59,598 | 66.4 | 94 | 1 | HM152764 | Pope *et al.* 2011a |
| Apocalypse | K1 | 59,947 | 66.4 | 98 | 1 | MF668267 | unpublished |
| Atiba | K1 | 59,556 | 66.5 | 96 | 1 | MN234230 | unpublished |
| BarrelRoll | K1 | 59,672 | 66.6 | 95 | 1 | JN643714 | Pope *et al.* 2015 |
| BEEST | K1 | 59,906 | 66.6 | 95 | 1 | MH509444 | Pope *et al.* 2015 |
| Beezoo | K1 | 60,494 | 66.5 | 99 | 1 | MH371113 | unpublished |
| Bella96 | K1 | 60,746 | 66.1 | 97 | 1 | MF377440 | Anders *et al.* 2017a |
| Belladonna | K1 | 59,708 | 66.6 | 96 | 1 | MH697578 | unpublished |
| BGlluviae | K1 | 59,308 | 66.5 | 95 | 1 | MN908692 | unpublished |
| Biglebops | K1 | 56,454 | 67.1 | 77 | 0 | MH399770 | unpublished |
| Capricorn | K1 | 59,708 | 66.5 | 96 | 1 | MK112537 | unpublished |
| Chris | K1 | 62,067 | 67.2 | 100 | 1 | MT310860 | unpublished |
| CREW | K1 | 59,707 | 66.6 | 94 | 1 | KY380102 | Pope *et al.* 2015 |
| CrimD | K1 | 59,798 | 66.9 | 96 | 1 | HM152767 | Pope *et al.* 2011b |
| Curiosium | K1 | 61,222 | 65.6 | 103 | 1 | MN234226 | unpublished |
| Dalmuri | K1 | 59,708 | 66.5 | 96 | 1 | MH727544 | unpublished |
| Deby | K1 | 60,463 | 66.5 | 95 | 1 | MG962364 | Gaballa *et al.* 2019 |
| DrHayes | K1 | 60,526 | 66.2 | 97 | 1 | KX657795 | Anders *et al.* 2017b |
| Efra2 | K1 | 61,284 | 66.6 | 103 | 1 | MN234174 | unpublished |
| Emerson | K1 | 60,310 | 66.6 | 101 | 1 | KJ567045 | Pope *et al.* 2015 |
| Enkosi | K1 | 59,052 | 67.2 | 79 | 0 | KT281789 | unpublished |
| Geraldini | K1 | 59,818 | 66.6 | 96 | 1 | MN234182 | unpublished |
| Guanica15 | K1 | 60,974 | 66.6 | 102 | 1 | MN234201 | unpublished |
| HedwigODU | K1 | 59,812 | 66.6 | 95 | 1 | KX585253 | unpublished |
| Homura | K1 | 59,708 | 66.6 | 94 | 1 | MH536821 | Pope *et al.* 2015 |
| Inky | K1 | 59,708 | 66.5 | 95 | 1 | MN369746 | unpublished |
| JAWS | K1 | 59,749 | 66.6 | 94 | 1 | JN185608 | Pope *et al.* 2015 |
| Jeckyll | K1 | 59,708 | 66.6 | 96 | 1 | MF140412 | unpublished |
| Joy99 | K1 | 59,837 | 66.6 | 97 | 1 | MH536822 | Pope *et al.* 2015 |
| KiSi | K1 | 62,558 | 68.7 | 103 | 1 | MK376955 | unpublished |
| LastHope | K1 | 60,934 | 66.9 | 102 | 1 | MF140416 | Anders *et al.* 2017a |
| LaterM | K1 | 60,143 | 66.5 | 95 | 1 | MG962371 | Gaballa *et al.* 2019 |
| LeMond | K1 | 62,515 | 68.8 | 102 | 1 | MH910038 | unpublished |
| LilPharaoh | K1 | 56,167 | 67.1 | 78 | 0 | MF919518 | Gaballa *et al.* 2019 |
| LindNT | K1 | 60,053 | 66.8 | 93 | 1 | KX641264 | unpublished |
| MarkPhew | K1 | 62,153 | 67.9 | 102 | 1 | MT310859 | unpublished |
| Mdavu | K1 | 56,443 | 67.1 | 78 | 0 | MN586025 | unpublished |
| Murucutumbu | K1 | 60,609 | 66.7 | 95 | 1 | KM677211 | Pope *et al.* 2015 |
| Mynx | K1 | 60,055 | 66.6 | 96 | 1 | MH513977 | unpublished |
| Nibb | K1 | 62,293 | 67.6 | 102 | 1 | MK460246 | unpublished |
| Niklas | K1 | 60,989 | 68.5 | 97 | 1 | MK494119 | unpublished |
| Oscar | K1 | 62,437 | 68.8 | 103 | 1 | MH910039 | unpublished |
| Peanam | K1 | 61,041 | 68.5 | 98 | 1 | MF185722 | Anders *et al.* 2017a |
| Prithvi | K1 | 60,311 | 66.8 | 97 | 1 | MK016503 | unpublished |
| Ramen | K1 | 59,462 | 66.6 | 96 | 1 | MN234197 | unpublished |
| Rapunzel97 | K1 | 59,687 | 66.6 | 98 | 1 | MN234231 | unpublished |
| SamuelLPlaqson | K1 | 60,526 | 66.2 | 97 | 1 | KX657794 | Anders *et al.* 2017b |
| Scarlett | K1 | 62,306 | 68.7 | 102 | 1 | MH910042 | unpublished |
| SgtBeansprout | K1 | 56,439 | 67.1 | 78 | 0 | MH020245 | Gaballa *et al.* 2019 |
| Shaobing | K1 | 61,030 | 68.5 | 95 | 1 | MK310138 | unpublished |
| Slimphazie | K1 | 60,143 | 66.6 | 98 | 1 | MF140428 | Anders *et al.* 2017a |
| Spock | K1 | 59,709 | 66.5 | 95 | 1 | MN369742 | unpublished |
| Stinson | K1 | 59,918 | 66.6 | 96 | 1 | MZ355721 | this study |
| Sulley | K1 | 59,873 | 66.4 | 94 | 1 | MF919532 | Pope *et al.* 2015 |
| Tachez | K1 | 59,556 | 66.5 | 96 | 1 | MF140430 | unpublished |
| TreyKay | K1 | 60,311 | 66.8 | 97 | 1 | MF472892 | unpublished |
| Urkel | K1 | 60,526 | 66.2 | 97 | 1 | KX657796 | Anders *et al.* 2017b |
| Validus | K1 | 62,466 | 68.4 | 106 | 1 | KF713486 | Pope *et al.* 2015 |
| Veliki | K1 | 59,734 | 66.6 | 96 | 1 | MN234205 | unpublished |
| YoureAdopted | K1 | 59,504 | 66.6 | 95 | 0 | MK460247 | unpublished |
| Yunkel11 | K1 | 60,757 | 66.6 | 100 | 1 | MN234165 | unpublished |
| Zavala | K1 | 59,969 | 66.6 | 97 | 1 | MN234198 | unpublished |
| BoostSeason | K2 | 58,078 | 68.2 | 94 | 0 | MH834601 | unpublished |
| DismalFunk | K2 | 58,129 | 68.3 | 94 | 0 | MF140408 | unpublished |
| DismalStressor | K2 | 58,129 | 68.3 | 94 | 0 | MH727545 | unpublished |
| Findley | K2 | 58,150 | 68.3 | 94 | 0 | MF140411 | Saha *et al.* 2017 |
| Marcoliusprime | K2 | 58,129 | 68.2 | 92 | 0 | KX688047 | unpublished |
| Milly | K2 | 58,211 | 68.3 | 93 | 1 | KP027206 | Pope *et al.* 2015 |
| Mufasa | K2 | 58,065 | 68.2 | 93 | 1 | KT591490 | Pope *et al.* 2015 |
| TM4 | K2 | 52,797 | 68.1 | 89 | 0 | AF068845 | Ford *et al.* 1998 |
| ZoeJ | K2 | 57,315 | 68.5 | 92 | 0 | KJ510412 | Pope *et al.* 2015 |
| Hurricane | K3 | 61,318 | 67.1 | 98 | 0 | MF373841 | Saha *et al.* 2017 |
| Keshu | K3 | 61,251 | 67.3 | 101 | 0 | KP027199 | Pope *et al.* 2015 |
| MacnCheese | K3 | 61,567 | 67.3 | 99 | 0 | JX042579 | Pope *et al.* 2015 |
| Pixie | K3 | 61,147 | 67.3 | 100 | 0 | JF937104 | Pope *et al.* 2015 |
| ShedlockHolmes | K3 | 61,081 | 67.3 | 100 | 0 | KR080206 | Pope *et al.* 2015a |
| TBond007 | K3 | 61,145 | 67.3 | 97 | 0 | KX683428 | Saha *et al.* 2017 |
| Chancellor | K4 | 57,697 | 68.0 | 94 | 1 | MF140402 | Edgington *et al.* 2017 |
| Cheetobro | K4 | 57,253 | 68.0 | 92 | 1 | KJ944841 | Pope *et al.* 2015 |
| Eponine | K4 | 58,678 | 67.4 | 96 | 1 | MN945904 | unpublished |
| Fionnbharth | K4 | 58,076 | 68.0 | 94 | 1 | JN831653 | Pope *et al.* 2015 |
| JF1 | K4 | 57,990 | 67.9 | 94 | 1 | MT310882 | Crawford *et al.* 1981 |
| Malthus | K4 | 57,802 | 67.9 | 95 | 1 | MN369761 | unpublished |
| MissDaisy | K4 | 54,464 | 67.6 | 89 | 0 | MK524485 | unpublished |
| Mitti | K4 | 57,895 | 68.0 | 94 | 1 | KY087992 | Edgington *et al.* 2017 |
| Patt | K4 | 54,611 | 67.6 | 88 | 0 | MK524488 | unpublished |
| Reptar3000 | K4 | 54,601 | 67.6 | 88 | 1 | MH926058 | Salisbury *et al.* 2019 |
| SamScheppers | K4 | 58,351 | 67.6 | 94 | 0 | MH051258 | Pope *et al.* 2015 |
| Slarp | K4 | 57,256 | 68.0 | 93 | 1 | KT361920 | Pope *et al.* 2015 |
| Taquito | K4 | 58,390 | 67.5 | 94 | 1 | KX621007 | Pope *et al.* 2015 |
| Wintermute | K4 | 58,046 | 68.0 | 94 | 1 | MF140435 | Edgington *et al.* 2017 |
| AlleyCat | K5 | 62,112 | 65.3 | 99 | 1 | MF185717 | King *et al.* 2017 |
| Collard | K5 | 61,395 | 65.6 | 94 | 1 | MH651171 | Pope *et al.* 2015 |
| Edugator | K5 | 63,344 | 65.3 | 92 | 1 | MF185719 | King *et al.* 2017 |
| Gengar | K5 | 61,626 | 65.0 | 95 | 1 | KX636165 | Pope *et al.* 2015 |
| Guillsminger | K5 | 63,153 | 65.0 | 95 | 1 | MF185720 | King *et al.* 2017 |
| Kratio | K5 | 62,738 | 65.7 | 99 | 1 | KM923971 | Pope *et al.* 2015 |
| Larva | K5 | 62,991 | 65.3 | 96 | 1 | JN243855 | Pope *et al.* 2015 |
| Leston | K5 | 61,808 | 64.9 | 94 | 1 | MH051255 | unpublished |
| OkiRoe | K5 | 62,661 | 64.9 | 97 | 1 | KJ567042 | Pope *et al.* 2015 |
| Omnicron | K5 | 61,511 | 64.0 | 95 | 1 | KM363596 | Pope *et al.* 2015 |
| Paola | K5 | 61,535 | 65.0 | 92 | 1 | MG962374 | Gaballa *et al.* 2019 |
| Psycho | K5 | 62,110 | 65.3 | 97 | 1 | MW435854 | unpublished |
| Rando14 | K5 | 59,925 | 64.3 | 91 | 1 | MH697592 | unpublished |
| Thyatira | K5 | 63,874 | 64.6 | 96 | 1 | MH576966 | unpublished |
| Waterfoul | K5 | 61,248 | 64.9 | 95 | 1 | KX585251 | Jackson *et al*. 2016 |
| Amgine | K6 | 62,236 | 66.4 | 97 | 0 | MF324915 | Anders *et al.* 2017a |
| Amohnition | K6 | 61,761 | 67.2 | 94 | 1 | MF140398 | Anders *et al.* 2017a |
| Bryler | K6 | 57,666 | 66.1 | 92 | 2 | MN369762 | unpublished |
| Cain | K6 | 60,813 | 66.3 | 100 | 2 | MF324913 | Anders *et al.* 2017a |
| DarthP | K6 | 61,594 | 67.2 | 94 | 1 | MF140406 | Anders *et al.* 2017a |
| Ekdilam | K6 | 61,772 | 67.4 | 92 | 0 | MN234199 | unpublished |
| Ellie | K6 | 61,945 | 67.0 | 96 | 0 | MT723940 | unpublished |
| Hammy | K6 | 61,812 | 67.2 | 94 | 1 | KY087993 | Anders *et al.* 2017a |
| Krueger | K6 | 60,321 | 66.5 | 100 | 1 | MF324914 | Anders *et al.* 2017a |
| Marshawn | K6 | 61,464 | 68.1 | 95 | 1 | MN284895 | unpublished |
| PhelpsODU | K6 | 56,580 | 66.1 | 90 | 2 | MF324909 | Anders *et al.* 2017a |
| Phrank | K6 | 61,109 | 66.2 | 101 | 2 | MF324912 | Anders *et al.* 2017a |
| SirPhilip | K6 | 61,882 | 66.7 | 97 | 1 | MF324911 | Anders *et al.* 2017a |
| Unicorn | K6 | 61,208 | 66.2 | 102 | 4 | MF324908 | Anders *et al.* 2017a |
| Ximenita | K6 | 61,027 | 66.7 | 102 | 1 | MN945901 | unpublished |
| Yuna | K6 | 62,192 | 67.4 | 99 | 1 | MN234176 | unpublished |
| Aminay | K7 | 60,430 | 67.8 | 105 | 0 | MH509442 | unpublished |

**Table S2. *Mycobacteria* included in the comparative analyses.**

| ***Mycobacterium*** | **# Genes** | **Length (kb)** | **GC-content** | **Accession #** | **Reference** |
| --- | --- | --- | --- | --- | --- |
| *M. abscessus* | 4,957 | 4,618 | 64.2 | CP004374 | Kim *et al.* 2103 |
| *M. africanum* | 4,069 | 4,493 | 65.1 | CP014617 | Hurtado *et al.* 2016 |
| *M. avium* | 3,935 | 3,981 | 69.3 | AE016958 | Li *et al.* 2005 |
| *M. bovis* | 3,952 | 3,972 | 65.6 | AM408590 | Brosch *et al.* 2007 |
| *M. canetti* | 4,139 | 4,482 | 65.6 | HE572590 | Bentley *et al.* 2012 |
| *M. chelonae* | 4,943 | 5,061 | 64 | CP050145 | Gu *et al.* 2020 |
| *M. fortuitum* | 6,023 | 6,255 | 66.2 | CP011269 | Costa *et al.* 2015 |
| *M. gilvum* | 5,139 | 5,077 | 67.9 | CP002385 | Kallimanis *et al.* 2011 |
| *M. intracellulare* | 5,143 | 4,936 | 68.1 | CP003322 | Kim *et al.* 2012 |
| *M. leprae* | 1,604 | 1,620 | 57.8 | AL450380 | Cole *et al.* 2001 |
| *M. marinum* | 5,422 | 5,973 | 65.2 | CP000854 | Stinear *et al.* 2008 |
| *M. smegmatis* | 6,692 | 6,508 | 67.4 | CP001663 | Deshayes *et al.* 2007 |
| *M. tuberculosis* | 3,935 | 3,981 | 65.6 | AL123456 | Cole *et al.* 1998 |
| *M. ulcerans* | 4,159 | 4,074 | 65.4 | CP000325 | Stinear *et al.* 2007 |

**Table S3. Prophages contained within putative mycobacterial host genomes.**

| ***Mycobacterium*** | **Region** | **Completeness** | **Mycobacteriophage (sub-cluster)** |
| --- | --- | --- | --- |
| *M. abscessus* | 1 | **Intact** | 32HC (Z)  Benedict (A5)  Brujita (I1)  Carcharodon (N)  Che9c (I2)  Cornie (F5)  Cuke (AC)  Donovan (P1)  Doug (F1)  Dylan (O)  Giles (Q)  Halo (G1)  Phineas (P1)  Pipsqueaks (N)  Purky (P6)  Rem711 (Z)  SkinnyPete (N)  Sneeze (G1)  TChen (F4)  ThetaBob (F4)  Tortellini (P2)  UnionJack (A5) |
|  | 2 | Incomplete |  |
|  | 3 | **Intact** | **Adephagia (K1)^[[1]](#footnote-1)^**  ArcusAngelus (F1)  Babsiella (I1)  Baka (J)  Bernal13 (T)  Bongo (M1)  ByChance (F1)  Courthouse (J)  DeadP (F1)  Frankie (F1)  Gaia (X)  Godines (B2)  Kratio (K5)  **LastHope (K1)^[[2]](#footnote-2)^**  Llama (F1)  Loser (A2)  Melissauren88 (F1)  MichelleMyBell (N)  Milly (K2)  Minerva (J)  MooMoo (singleton)  Omega (J)  Omnicron (K5)  Optimus (J)  OwlsT2W (F1)  Patience (U)  Pipsqueaks (N)  Pumpkin (E)  Sbash (I2)  Sparky (singleton)  Squirty (F3)  SuperGrey (F1)  Taquito (K4)  ThetaBob (F4)  TM4 (K2)  Tortellini (P2)  Trouble (A1)  Zapner (F2) |
|  | 4 | Incomplete |  |
|  | 5 | **Intact** | **Amelie (K1)^[[3]](#footnote-3)^**  **Beezoo (K1)^[[4]](#footnote-4)^**  Benedict (A5)  Bipper (Y)  DS6A (singleton)  Gaia (X)  Jobu08 (A3)  Kratio (K5)  KristaRAM (F1)  Liefie (G1)  Llama (F1)  Lolly9 (L3)  Milly (K2)  MooMoo (singleton)  MosMoris (S)  Muddy (AB)  Omega (J)  Omnicron (K5)  Rebeuca (A10)  Rem711 (Z)  RhynO (A10)  Sarfire (A1)  SassyB (F1)  Send513 (R)  Taquito (K4)  TChen (F4)  ThulaThula (P5)  Tortellini (P2)  UnionJack (A5) |
| *M. africanum* | 1 | Incomplete |  |
|  | 2 | Incomplete |  |
| *M. avium* | 1 | Incomplete |  |
|  | 2 | Incomplete |  |
|  | 3 | Incomplete |  |
| *M. bovis* | 1 | Incomplete |  |
| *M. canetti* | 1 | Incomplete |  |
|  | 2 | Incomplete |  |
|  | 3 | Incomplete |  |
| *M. chelonae* | 1 | Incomplete |  |
|  | 2 | Incomplete |  |
| *M. fortuitum* | 1 | Incomplete |  |
|  | 2 | Incomplete |  |
| *M. gilvum* | 1 | Incomplete |  |
|  | 2 | Questionable |  |
|  | 3 | Questionable |  |
|  | 4 | Incomplete |  |
|  | 5 | Incomplete |  |
| *M. intracellulare* | 1 | Incomplete |  |
| *M. leprae* | – |  |  |
| *M. marinum* | 1 | Incomplete |  |
|  | 2 | **Intact** | **Amelie (K1)^[[5]](#footnote-5)^**  ArcusAngelus (F1)  Bipper (Y)  Cornie (F5)  Cuke (AC)  Gumball (D1)  Hawkeye (D2)  Indlulamithi (AC)  JC27 (A1)  Kingsley (F1)  Marshawn (K6)  Mendokysei (T)  Microwolf (A3)  Mozy (F1)  Phrann (N)  Piper2020 (F1)  Rem711 (Z)  Send513 (R)  Sneeze (G1)  Sparky (singleton)  ThulaThula (P5)  Veteran (F1) |
| *M. smegmatis* | 1 | Incomplete |  |
|  | 2 | Incomplete |  |
|  | 3 | Incomplete |  |
|  | 4 | **Intact** | *None* |
|  | 5 | Incomplete |  |
|  | 6 | Incomplete |  |
|  | 7 | Incomplete |  |
|  | 8 | Incomplete |  |
|  | 9 | Incomplete |  |
|  | 10 | Incomplete |  |
| *M. tuberculosis* | 1 | Incomplete |  |
|  | 2 | Incomplete |  |
|  | 3 | Incomplete |  |
| *M. ulcerans* | 1 | Incomplete |  |

**LITERATURE CITED**

Anders, K. R., N. Barekzi, A. A. Best, G. D. Frederick, D. V. Mavrodi, *et al*., 2017a Genome sequences of mycobacteriophages Amgine, Amohnition, Bella96, Cain, DarthP, Hammy, Krueger, LastHope, Peanam, PhelpsODU, Phrank, SirPhilip, Slimphazie, and Unicorn.  Genome Announc. 5(49): e01202–17.

Anders, K. R. , A. M. Murphy, W. F. Ettinger, D. Kempthorne, C. Kittridge, *et al.*, 2017b Genome sequences of cluster K mycobacteriophages DrHayes, Urkel, and SamuelLPlaqson. Genome Announc. 5(16): e01388–16.

Bentley, S. D., I. Comas, J. M. Bryant, D. Walker, N. H. Smith, *et al*., 2012 The genome of *Mycobacterium africanum* West African 2 reveals a lineage-specific locus and genome erosion common to the *M. tuberculosis* complex. PLoS Negl. Trop. Dis. 6(2): e1552.

Brosch, R., S. V. Gordon, T. Garnier, K. Eiglmeier, W. Frigui, *et al*., 2007 Genome plasticity of BCG and impact on vaccine efficacy. Proc. Natl. Acad. Sci. U. S. A. 104(13): 5596–5601.

Cole, S. T., R. Brosch, J. Parkhill, T. Garnier, C. Churcher, *et al*., 1998 Deciphering the biology of *Mycobacterium tuberculosis* from the complete genome sequence. Nature. 393(6685): 537–544.

Cole, S. T., K. Eiglmeier, J. Parkhill, K. D. James, N. R. Thomson, *et al*., 2001 Massive gene decay in the leprosy bacillus. Nature. 409(6823): 1007–1011.

Costa, K. C., M. Bergkessel, S. Saunders, J. Korlach, D. K. Newman, 2015 Enzymatic degradation of phenazines can generate energy and protect sensitive organisms from toxicity. mBio. 6(6): e01520-15.

Crawford, J. T., J. K. Fitzhugh, J. H. Bates, 1981 Phage typing of the Mycobacterium avium-intracellulare-scrofulaceum complex. Am. Rev. Respir. Dis. 124(5): 559–562.

Deshayes, C., E. Perrodou, S. Gallien, D. Euphrasie, C. Schaeffer, *et al*., 2007 Interrupted coding sequences in *Mycobacterium smegmatis*: authentic mutations or sequencing errors? Genome Biol. 8(2): R20.

Edgington, N. P., S. M. Voshell, V. C. Ware, F. F. Akoto, A. A. Alhout, *et al.,* 2017 Genome sequences of Chancellor, Mitti, and Wintermute, three subcluster K4 phages isolated using *Mycobacterium smegmatis* mc^2^155. Genome Announc. 5(45): e01070–17.

Ford, M. E., C. Stenstrom, R. W. Hendrix, G. F. Hatfull, 1998 Mycobacteriophage TM4: genome structure and gene expression. Tuber. Lung. Dis. 79(2): 63–73.

Gaballa, J. M., K. Dabrian, R. Desai, R. Ngo, D. Park, *et al.*, 2019 Genome sequences of cluster K mycobacteriophages Deby, LaterM, LilPharaoh, Paola, SgtBeansprout, and Sulley. Microbiol. Resour. Announc. 8(2): e01481–18.

Gu, C. H., C. Zhao, C. Hofstaedter, P. Tebas, L. Glaser, *et al*., 2020 Investigating hospital *Mycobacterium chelonae* infection using whole genome sequencing and hybrid assembly. PLoS One. 15(11): e0236533.

Hatfull, G. F., 2012a. Complete genome sequences of 138 mycobacteriophages. J. Virol. 86(4): 2382–4.

Hurtado, U. A., J. S. Solano, A. Rodriguez, J. Robledo, F. Rouzaud, 2016 Draft genome sequence of a *Mycobacterium africanum* clinical isolate from Antioquia, Colombia. Genome Announc. 4(3): e00486-16.

Jackson, P. N., E. K. Embry, C. O. Johnson, T. L. Watson, S. K. Weast, *et al.*, 2016 Genome sequence of mycobacterium phage Waterfoul. Genome Announc. 4(6): e01281–16.

Kallimanis, A., E. Karabika, K. Mavromatis, A. Lapidus, K. M. Labutti, *et al.*, 2011 Complete genome sequence of *Mycobacterium sp*. strain (Spyr1) and reclassification to *Mycobacterium gilvum* Spyr1. Stand Genomic Sci. 5(1): 144–153.

Kim, B. J., B. S. Choi, J. S. Lim, I. Y. Choi, J. H. Lee, *et al.,* 2012 Complete genome sequence of *Mycobacterium intracellulare* strain ATCC 13950(T). J. Bacteriol. 194(10): 2750.

Kim, B. J., B. R. Kim, S. H. Hong, S. H. Seok, Y. H. Kook, *et al.*, 2013 Complete genome sequence of *Mycobacterium massiliense* clinical strain Asan 50594, belonging to the type II genotype. Genome Announc. 1(4): e00429-13.

King, A. R., T. M. Slowan-Pomeroy, J. E. Thomas, T. Ahmed, K. L. Alexander, *et al.*, 2017 Genome sequences of subcluster K5 mycobacteriophages AlleyCat, Edugator, and Guillsminger. Genome Announc. 5(45): e01122–17.

Li, L., J. P. Bannantine, Q. Zhang, A. Amonsin, B. J. May, *et al.*, 2005 The complete genome sequence of *Mycobacterium avium* subspecies paratuberculosis. Proc. Natl. Acad. Sci. U. S. A. 102(35): 12344–12349.

Pope, W. H., J. T. Carter, K. L. Dasher, M. C. Haynberg, A. Reddi, *et al*., 2015a Genome sequence of a newly isolated mycobacteriophage, ShedlockHolmes. Genome Announc. 3(3): e00597–15.

Saha, S. W. Yan, J. M. Washington, W. B. Davis, W. B. Barbazuk, *et al.,* 2017 Genome sequences of mycobacteriophages Findley, Hurricane, and TBond007. Genome Announc. 5(45): e01123–17.

Salisbury, A., E. Cassin, K. Ayala-Pineda, N. Barroga, V Cadiz, *et al*., 2019 Complete genome sequences of *Mycobacterium smegmatis* phages Chewbacca, Reptar3000, and Riparian, isolated in Las Vegas, Nevada. Microbiol. Resour. Announc. 8(6): e01558–18.

Stinear, T. P., T. Seemann, P. F. Harrison, G. A. Jenkin, J. K. Davies, *et al*., 2008 Insights from the complete genome sequence of *Mycobacterium marinum* on the evolution of *Mycobacterium tuberculosis*. Genome Res. 18(5): 729–741.

Stinear, T. P., T. Seemann, S. Pidot, W. Frigui, G. Reysset, *et al.*, 2007 Reductive evolution and niche adaptation inferred from the genome of *Mycobacterium ulcerans*, the causative agent of Buruli ulcer. Genome Res. 17(2): 192–200.

1. e-value: 5.53E-05 [↑](#footnote-ref-1)
2. e-value: 2.32E-07 [↑](#footnote-ref-2)
3. e-value: 0 [↑](#footnote-ref-3)
4. e-value: 1.35E-06 [↑](#footnote-ref-4)
5. e-value: 8.12E-05 [↑](#footnote-ref-5)
